# Meiosis completion and various sperm responses lead to unisexual and sexual reproduction modes in one clone of polyploid *Carassius gibelio*

**DOI:** 10.1038/srep10898

**Published:** 2015-06-04

**Authors:** Jun Zhang, Min Sun, Li Zhou, Zhi Li, Zhen Liu, Xi-Yin Li, Xiao-Li Liu, Wei Liu, Jian-Fang Gui

**Affiliations:** 1 State Key Laboratory of Freshwater Ecology and Biotechnology, Institute of Hydrobiology, Chinese Academy of Sciences, University of Chinese Academy of Sciences, Wuhan 430072, China; 2 School of Life Science, Shanxi University, Taiyuan 030006, China

## Abstract

Unisexual polyploid vertebrates are commonly known to reproduce by gynogenesis, parthenogenesis, or hybridogenesis. One clone of polyploid *Carassius gibelio* has been revealed to possess multiple modes of unisexual gynogenesis and sexual reproduction, but the cytological and developmental mechanisms have remained unknown. In this study, normal meiosis completion was firstly confirmed by spindle co-localization of β-tubulin and Spindlin. Moreover, three types of various nuclear events and development behaviors were revealed by DAPI staining and BrdU-incorporated immunofluorescence detection during the first mitosis in the fertilized eggs by three kinds of different sperms. They include normal sexual reproduction in response to sperm from the same clone male, typical unisexual gynogenesis in response to sperm from the male of another species *Cyprinus carpio*, and an unusual hybrid-similar development mode in response to sperm from another different clone male. Based on these findings, we have discussed cytological and developmental mechanisms on multiple reproduction modes in the polyploid fish, and highlighted evolutionary significance of meiosis completion and evolutionary consequences of reproduction mode diversity in polyploid vertebrates.

Sexual reproduction is universal in animals even though a twofold cost relative to unisexual or asexual reproduction is often attributed to the production of males and genome dilution^1^,^2^,^3^. The reason behind sexual reproduction success has been believed to be evolutionary advantage of recombination involving exchange of genetic information between individuals^4^, through which recombinational genetic variation and new genotypic combinations facilitate removal of deleterious mutations and provide great adaptive potentials^5^. In contrast, unisexual or asexual lineages were formerly thought to be short-lived on an evolutionary time scale because of recombination absence and deleterious mutation accumulation^5^,^6^,^7^. However, the unexpectedly ancient age of more than million years has been revealed in some unisexual vertebrates by mutation rate calculations of mitochondrial and nuclear genome sequences, and the oldest unisexual vertebrate *Ambystoma* salamander has been estimated to be up to 5 million years^8^,^9^,^10^. Therefore, the paradox between deleterious mutation accumulation and ancient age origin has been oppugned by many evolutionary geneticists^8^,^9^,^10^,^11^,^12^.

Since the first unisexual fish, the Amazon molly *Poecilia formosa*, was found in 1932^13^, about 90 all-female unisexual complexes have been reported in fish, amphibians and reptiles^6^,^14^. These unisexual animals have been demonstrated to reproduce by gynogenesis, hybridogenesis, parthenogenesis, or kleptogenesis^8^,^10^,^15^,^16^, but how to restore the exact chromosome number and genome ploidy level and how to maintain the genetic diversity for adapting to variable environments have remained unclear. Interestingly, a polyploid fish *Carassius gibelio* was discovered to possess abundant genetic diversity and multiple modes of unisexual gynogenesis and sexual reproduction^8^,^17^. *Carassius gibelio*, commonly known as Prussian carp, silver crucian carp or gibel carp, also named as a subspecies *Carassius auratus gibelio* of *Carassius auratus*^8^,^18^,^19^, was preliminarily found to be able to reproduce by unisexual reproduction of gynogenesis early in the last century^8^. It has diverse karyotypes with 156 or 162 chromosomes^20^, and genome reshuffling, chromosome and chromosomal fragment incorporation have occurred by manipulation or mating between various clones^21^. In comparison with domestic goldfish with 100 chromosomes, its two rounds of polyploidy origin and occurrence of hexaploid have been confirmed by 5 S rDNA fluorescence *in situ* hybridization (FISH), individual chromosome painting, and evolutionary history analysis of two divergent *Dmrt1* genes^22^,^23^,^24^. And, numerous different clones and genetic diversity have been identified in natural populations by serum transferrin phenotypes, RAPD (random amplification polymorphism of DNA) and SCAR markers, microsatellite markers, transferrin allele polymorphism, and mtDNA control region sequences^25^,^26^,^27^,^28^,^29^. Significantly, a minor but significant portion of males with identical genetic background have been found in natural populations from China^8^,^19^,^30^, Russia^31^, Greece^32^ and Croatia^33^, and multiple reproduction modes, including sexual reproduction, unisexual gynogenesis, or even androgenesis, have been demonstrated to coexist in one clone (clone D) of the polyploid fish^8^,^17^,^34^. However, one significant biological puzzle, i.e. how to restore and maintain the exact chromosome number and genome ploidy level in the different reproduction offspring, has to be solved from cytological and developmental investigations. In the past 80 years, several different mechanisms have been suggested, and they are various between and even within species in different unisexual animals^35^,^36^. A premeiotic endomitosis was proposed in gynogenetic vertebrates, such as *Ambystoma* salamanders^37^, *Poeciliopsis* fish^38^, *Aspidoscelis* lizards^39^, and *Misgurnus* loaches^40^, while another distinct cytological mechanism of apomixes or ameiosis (absence of meiosis) was characterized in Amazon molly *Poecilia formosa* and the triploid hybrids^41^. Additionally, an automictic reproduction mechanism in which random fusion of meiotic products occurs after the second meiosis was also suggested in interspecific hybrids of poeciliid fish^42^. In the polyploid *Carassius gibelio*, a similar cytological mechanism of ameiosis was previously hypothesized to explain the mother’s ploidy level maintenance in Russia, China and Japan^19^,^43^,^44^, in which the correct ploidy level was restored or maintained by inhibiting the first polar body extrusion. Along with discovery of the coexisting modes of unisexual and sexual reproductions, however, all above mechanisms are unable to explain the reason why the two kinds of offspring resulted from different reproduction modes can restore the exact chromosome number and genome ploidy level. Through a series of studies for more than 10 years, our laboratory has identified some important genes involved in oogenesis, oocyte maturation and egg fertilization from the polyploid fish^45^,^46^,^47^,^48^,^49^, and found that a significant maternal-effect factor Spindlin, which localizes on meiotic spindle^49^, might be a useful molecular marker for elaborating regulative mechanism of the unique multiple reproduction modes. In this study, we aim to utilize the spindle localization of β-tubulin and Spindlin to trace the meiosis process, and attempt to use DAPI staining as well as BrdU incorporation and immunofluorescence detection to reveal dynamic development progresses and early embryonic cleavage changes between different reproduction modes.

## Results

### Oocyte maturation and normal meiosis completion

**Figure 1:**
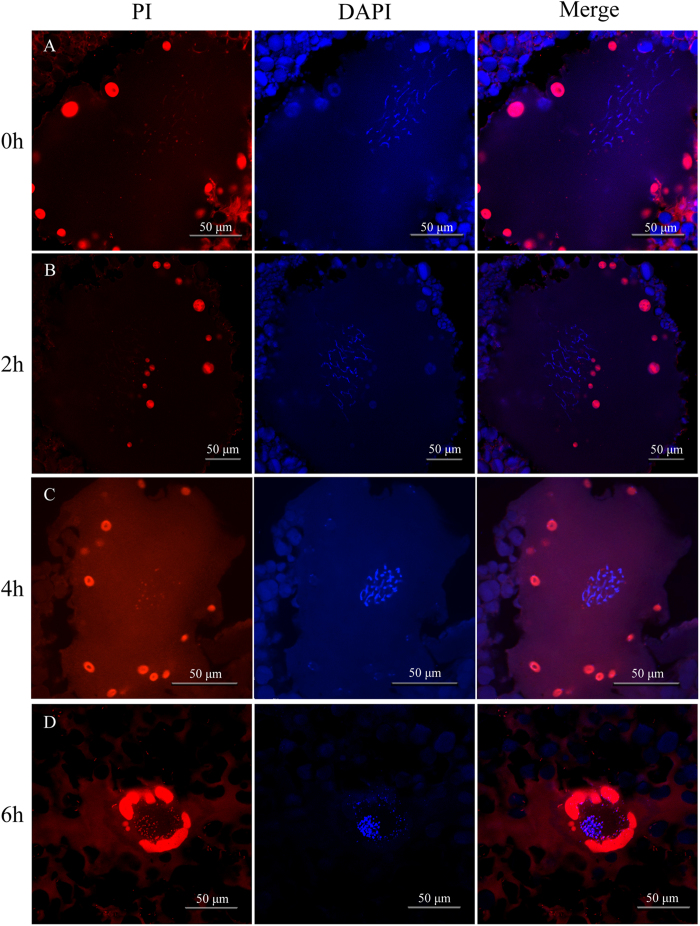
Meiotic chromosome dynamics in germinal vesicle breakdown (GVBD) process. The cryo-sections of previous stage oocytes at 0 h (**A**), 2 h (**B**), 4 h (**C**), and 6 h (**D**) after the hormone induction were stained by PI red fluorescence for the periphery nucleoli and DAPI blue fluorescence for meiotic chromosomes. The inducing time is shown on the left.

**Figure 2:**
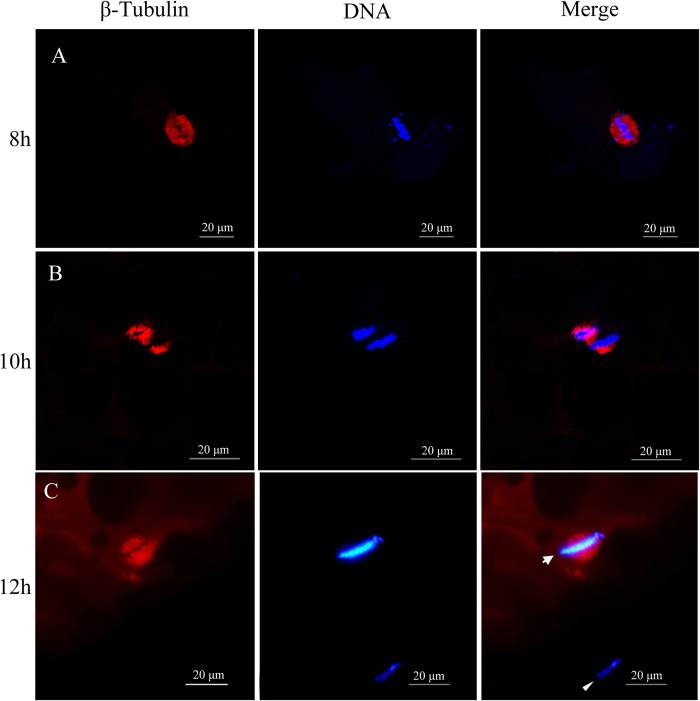
The first meiosis completion and the first polar body extrusion. The cryo-sections of late stage oocytes and mature egg at 8 h (**A**), 10 h (**B**), and12 h (**C**) after hormone induction were immunostained by β-tubulin-specific antibody for meiotic spindle and stained by DAPI for chromosomes respectively. Arrow indicates egg nucleus arrested at the second meiosis metaphase, and arrowhead indicates the first polar body.

To investigate whether normal meiosis was completed in clone D of *Carassius gibelio*, we collected different stage maturing oocytes and mature eggs from the artificial induction spawning females. Firstly, the cryo-sections of previous stage oocytes were stained by PI red fluorescence and DAPI blue fluorescence to recognize and trace meiotic chromosome dynamics and germinal vesicle breakdown (GVBD) process. As shown in Fig. 1, the periphery nucleoli around GV show strong red fluorescence stained by PI owing to their abundant RNA, and the DAPI-stained meiotic chromosomes clarify dynamic changes of prophase chromosomes from pachytene at induction beginning (Fig. 1A) and 2 h after the hormone injection (Fig. 1B) to diakinesis at 4 h (Fig. 1C) and 6 h (Fig. 1D). Significantly, along with the inducing maturation progress, GV migration occurs in 2 h and 4 h oocytes (Fig. 1B,C), and GVBD takes place in 6 h oocytes (Fig. 1D).

Subsequently, the β-tubulin-specific mouse antibody was used to localize meiotic spindle on the cryo-sections of late stage oocytes and mature eggs by showing red immunofluorescence with Rhodamine-conjugated goat anti-mouse antibody, and the DAPI-stained blue chromosomes were simultaneously employed to present dynamic course of homologous chromosome separation and the first polar body extrusion. As shown in Fig. 2, the β-tubulin-specific antibody-labeled microtubules are assembled into meiotic spindle in 8 h induction oocytes (Fig. 2A), half of homologous chromosomes are segregated into the first polar body, and the remaining chromosomes are captured by a second meiotic spindle in 10 h oocytes (Fig. 2B). Moreover, the first polar body is extruded, and mature eggs are arrested in the second meiosis metaphase for awaiting fertilization (Fig. 2C). The data indicate that the first meiotic division has completed in the clone along with oocyte maturation.

### Meiotic pachytene bivalents in GVs

**Figure 3:**
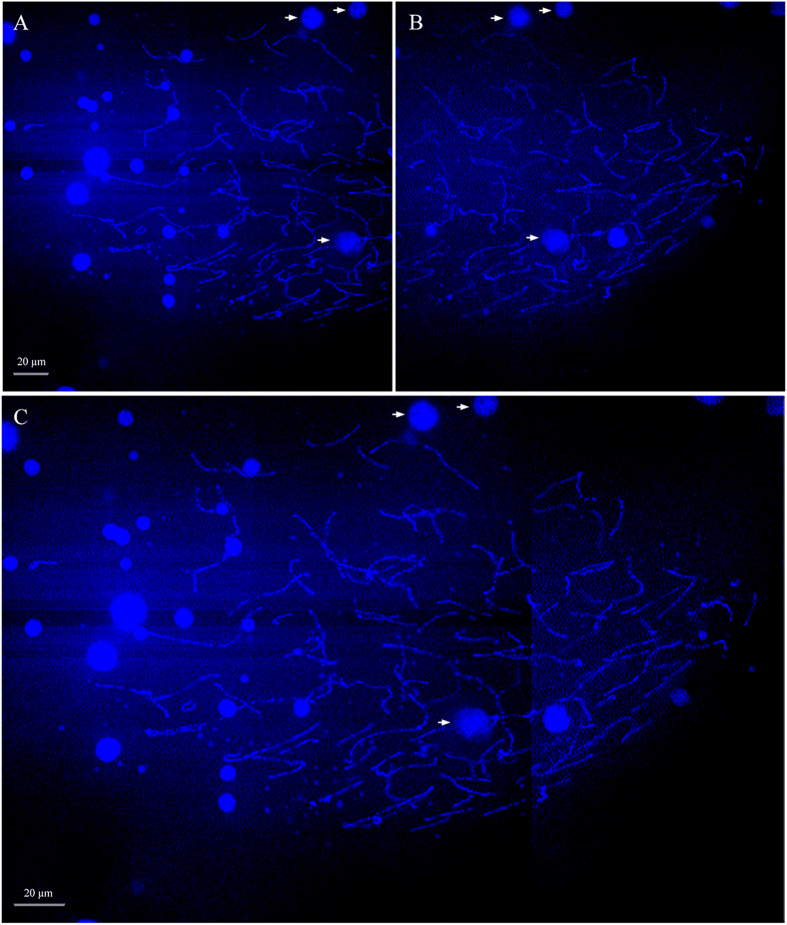
DAPI-stained meiotic chromosome spread in one pachytene GV. Since the spread was too big, two images (**A**, **B**) were taken for each GV spread by 63 × oil objective lens under two photon of Laser scanning confocal microscope. Then, they were merged into a complete meiotic pachytene bivalent configuration (**C**).

To confirm normal meiosis completion, we isolated GVs from full grown oocytes of clone D and prepared the DAPI-stained meiotic chromosome spread to determine numbers of meiotic chromosomes in each GV. As shown in Fig. 3, a total of 81 distinguishable pachytene bivalents are observed in the typical meiotic chromosome spread. A total of ten GVs were detected, and each GV was found to contain 75 to 81 pachytene bivalents. Therefore, normal meiosis completion has occurred because the number of meiotic pachytene bivalents is just about half of the somatic 162 chromosomes^20^.

### The first polar body extrusion

**Figure 4:**
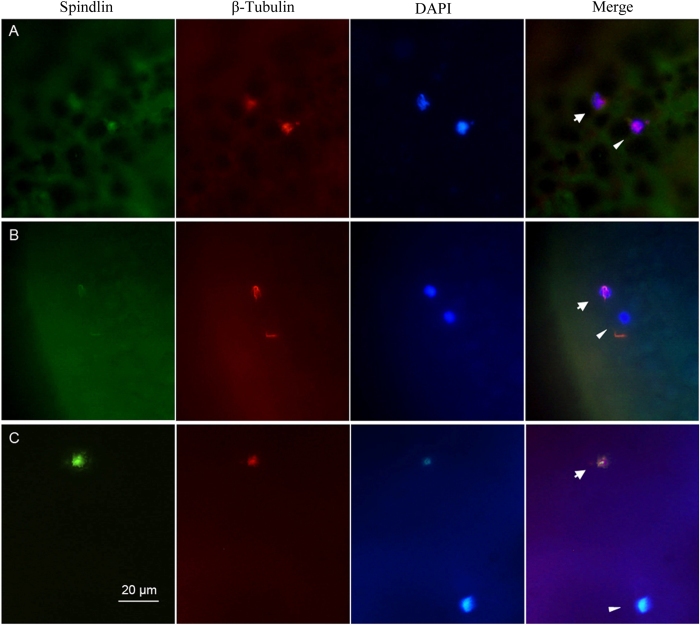
The detailed procedures of the first polar-body extrusion. Maturing oocytes (**A**, **B**) and mature eggs (**C**) were triple labeled by green immunofluorescence for spindlin, red immunofluorescence for β-tubulin, and DAPI blue fluorescence for chromosomes at late stage of the first meiotic division. Arrows indicate the egg nucleus, and arrowheads indicate the first polar-body.

To further confirm the above observation about meiosis completion, we used Spindlin-specific and β-tubulin-specific antibodies to co-localize meiotic spindles because previous study had demonstrated that Spindlin co-localized with β-tubulin on the meiotic spindles^49^, and thereby to trace the process of the first polar body extrusion in maturing oocytes and mature eggs by whole-mount immunofluoresence. As shown in Fig. 4, the detailed procedures of the first polar-body extrusion are recognized by triple fluorescence labeling of green immunofluorescence for Spindlin, red immunofluorescence for β-tubulin, and DAPI blue fluorescence for chromosomes at late stage of the first meiotic division. Firstly, the first polar-body is extruded from maturing oocyte, and Spindlin and β-tubulin are co-localized with inner and outer spindles and chromosomes at that time (Fig. 4A). Then, Spindlin and β-tubulin are detached from the extruded polar body along with further maturing of the oocyte (Fig. 4B). Finally, the extruded spindle as well as the associated Spindlin and β-tubulin are degraded, and only the DAPI-stained blue chromosome rudiment is visible from the first polar body in mature egg (Fig. 4C). Significantly, the inner Spindlin green immunofluorescence and β-tubulin red immunofluorescence in mature egg are stronger than that in maturing oocytes, and are still associated with chromosomes (Fig. 4C), implicating that a second meiotic spindle is assembled around the remaining chromosomes.

### The second polar body extrusion in the fertilized eggs

**Figure 5:**
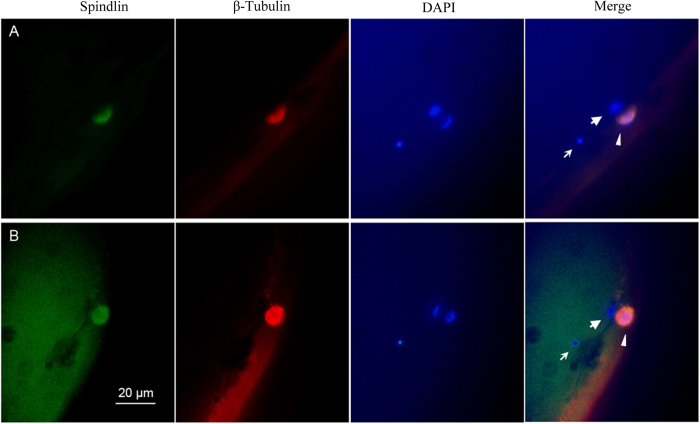
The second polar body extrusion in the fertilized eggs by same clone sperm (A) and by another species common carp sperm (B). The two fertilized eggs at 10 min after fertilization were triple labeled by green immunofluorescence for spindlin, red immunofluorescence for β-tubulin, and DAPI blue fluorescence for chromosomes. Arrows show the female nucleus. Arrowheads show the second polar-body. Small arrows indicate sperm nucleus.

Moreover, we used the same whole-mount triple fluorescence localization to label the second meiosis completion process in the fertilized eggs. As shown in Fig. 5, the second polar-body extrusion is also observed at 10 min after fertilization, no matter whether the inseminated sperm is from same clone (Fig. 5A) or from another species *Cyprinus carpio* (Fig. 5B). In comparison with mature eggs, the inner spindle and the associated Spindlin and β-tubulin cannot be recognized in the fertilized eggs by Spindlin and β-tubulin antibodies (Fig. 5), because fertilization has blockaded the plasma membrane passages for antibody entrance^45^.

### Differential nuclear events and development behaviors of the fertilized eggs in response to three kinds of different sperms

**Figure 6:**
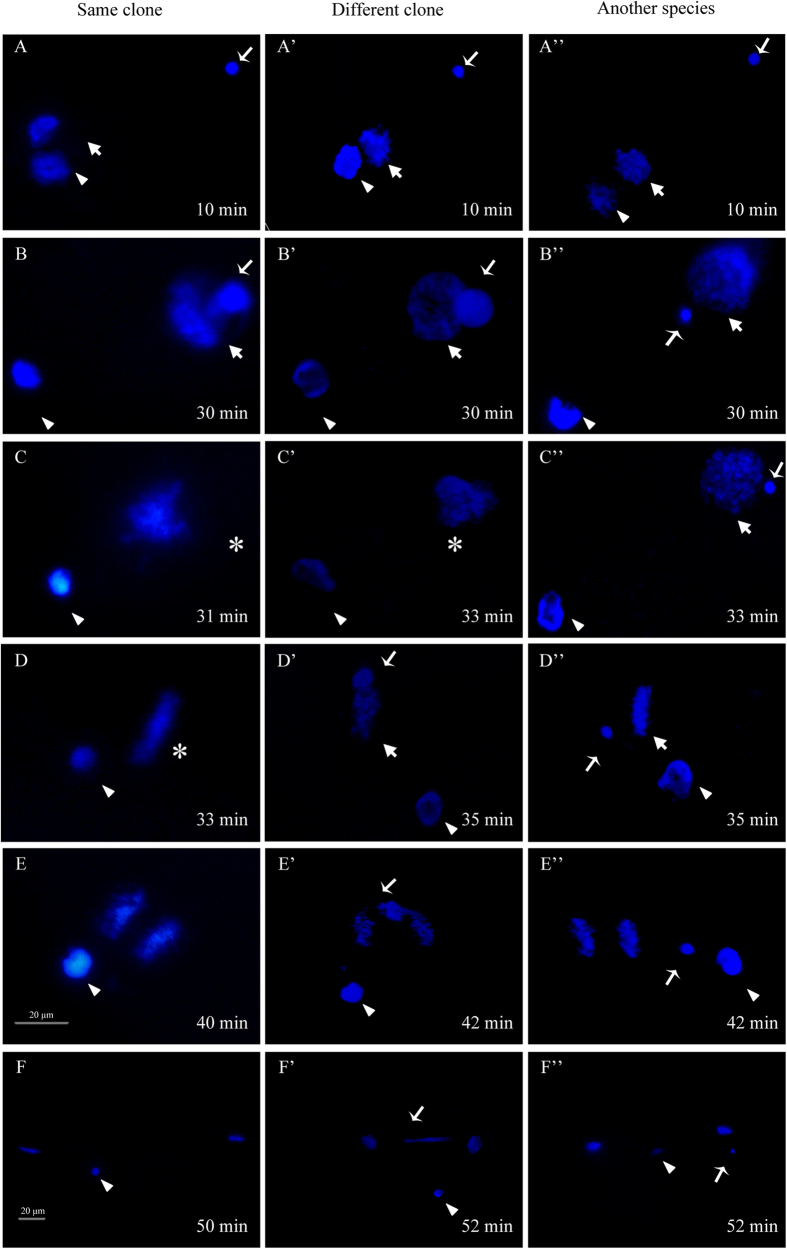
DAPI-stained nuclear events and development behaviors during the first mitosis from 10 min after sperm enter to the first mitosis completion. The eggs were respectively fertilized by the sperm from the same clone male (same clone) (**A**–**F**), by the sperm from different clone male (different clone) (**A'**–**F'**), and by the sperm from the male of another species *Cyprinus carpio* (**A''**–**F''**). The corresponding time (min) is shown on the right corner. Arrowheads indicate the second polar-body. Thick arrows indicate female pronucleus. Thin arrows indicate sperm nucleus or male pronucleus. Asterisks show the zygote nucleus after two pronucleus fusion in eggs fertilized by homologous sperm.

The above findings implicate that the clone D completes meiosis and produces normal reduced eggs. However, we have still unknown the mechanism how the meiotic completion eggs restore the exact chromosome number and genome ploidy level in responses to multiple modes including unisexual gynogenesis and sexual reproduction. For this purpose, we firstly used DAPI staining to observe nuclear event and development behavior difference of the fertilized eggs in response to three kinds of different sperms. As shown in Fig. 6, as the eggs are fertilized by the sperm from the same clone males (same clone), several classical nuclear events of sexual reproduction, such as sperm nucleus swelling and second polar-body extrusion before 10 min (Fig. 6A), migration and fusion of female pronucleus and male pronucleus from 10 min to 30 min (Fig. 6A,B), zygote formation of female pronuclus and male pronucleus fusion at 31 min (Fig. 6C), the first mitosis metaphase at 33 min (Fig. 6D), anaphase at 40 min (Fig. 6E) and 2-cell embryo formation after the first mitosis completion at about 50 min (Fig. 6F), are observed from the early embryos.

As the eggs are fertilized by the sperm from different clone males of *Carassius gibelio* (different clone), some early nuclear events, including sperm nucleus swelling and second polar-body extrusion before 10 min (Fig. 6A'), migration and fusion of female pronucleus and male pronucleus from 10 min to 30 min (Fig. 6A'), zygote formation of female pronuclus and male pronucleus fusion at 33 min (Fig. 6C'), are basically identical to that in the corresponding column eggs fertilized by the same clone sperm. Before the first mitosis initiates at 35 min, however, an unusual nucleus behavior occurs in the fertilized eggs, in which the chromosomes of male pronucleus fail to integrate the first zygotic mitosis (Fig. 6D'). As the mitosis progresses, the maternal chromosomes move toward the poles, whereas the divorced chromosomes obviously delay during the mitosis at 42 min (Fig. 6E') and finally form a typical chromatin bridge connecting the daughter cells after the first mitosis completion at 52 min (Fig. 6F').

As the eggs are inseminated by the sperm from males of another species *Cyprinus carpio*, the second polar-body extrusion occurs before 10 min (Fig. 6A''), but the entered sperm nucleus and female pronucleus undergo absolutely different nuclear events and behavior changes in the corresponding stages of the first mitosis. The sperm nucleus has been kept as the condensed status throughout the whole process from the initial enter to 2-cell embryo formation (Fig. 6A''–F''), and only the female nucleus accomplishes all steps of the first mitosis including female pronulcleus formation from 10 min to 33 min (Fig. 6A''–C''), the first mitosis metaphase at 35 min (Fig. 6D''), anaphase at 42 min (Fig. 6E'') and 2-cell embryo formation at about 52 min (Fig. 6F''), which is a typical procedure of gynogenesis^6^,^8^.

The above dynamic nucleus behavior and timing difference suggest that a differential genome replication and the associated development behavior change might have occurred in the fertilized eggs.

### BrdU incorporation-marked genome replication and development behavior difference in the fertilized eggs

**Figure 7:**
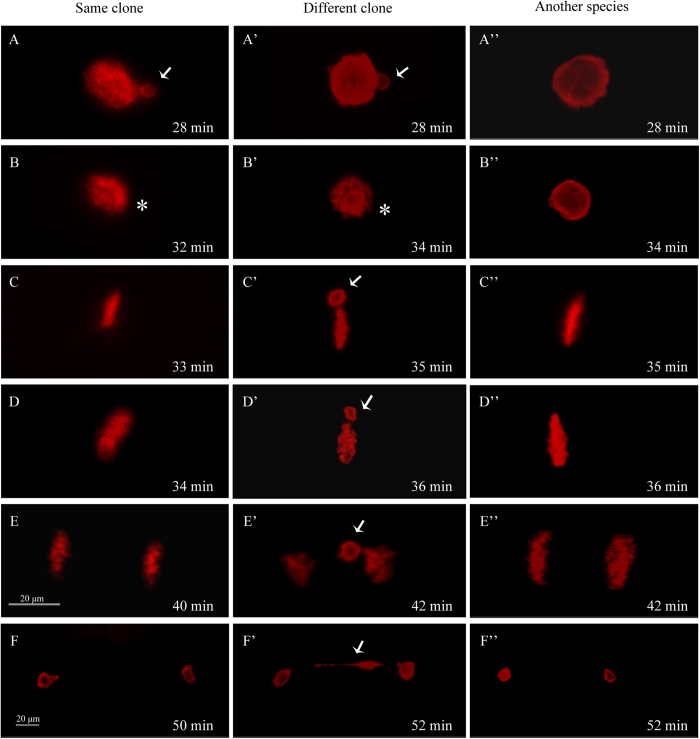
BrdU incorporation-marked genome replication and development behavior difference in the fertilized eggs by three kinds of different sperms. These different sperms include the sperm from the same clone male (**A**–**F**), the sperm from the different clone male (**A'**–**F'**), and the sperm from male of another species *Cyprinus carpio* (**A''**–**F''**). The corresponding time (min) is shown on the right corner. Thin arrows indicate the replicated sperm nucleus or male pronucleus. Asterisks show the zygote nucleus after two pronucleus fusion. In the activated eggs by the sperm from males of another species *Cyprinus carpio*, no sperm nucleus replication signal is visualized during the whole first mitosis (**A''**–**F''**).

To confirm the nucleus behavior differences and thereby to reveal the caused mechanism, we used BrdU incorporation and immunofluorescence detection method^49^ to trace precleavage genome replication status and the associated development behavior change in the fertilized eggs. As shown in Fig. 7, in the fertilized eggs by the sperm from the same clone males, both female pronucleus and male pronucleus undergo genome replication as they migrate and contact with each other at 28 min (Fig. 7A). And, the two replicating pronuclei combine and form zygote nucleus at 32 min (Fig. 7B). Then, zygote enters the first cleavage metaphase along with chromosome condensation at 33 min (Fig. 7C). Moreover, the chromosome segregation begins from 34 min (Fig. 7D) and reaches to telophase at about 40 min (Fig. 7E). Finally, the first cleavage completes and forms 2-cell embryo at 50 min (Fig. 7F). In the fertilized eggs by the sperm from the different clone males, both female pronucleus and male pronucleus also experience genome replication (Fig. 7A') and zygote formation from 28 min to 34 min (Fig. 7B'). However, when the replicated maternal chromosomes are aligned on metaphase plate at 35 min, the replicated male chromatin bubble is divorced from the maternal chromosomes (Fig. 7C'). Moreover, as the replicated maternal chromosomes move toward two poles from anaphase at 36 min (Fig. 7D'), telophase at 42 min (Fig. 7E') to the first mitosis completion at 52 min (Fig. 7F'), the divorced chromatin lags behind the maternal chromosomes (Fig. 7D'), lengthens (Fig. 7E'), and finally forms an typical chromatin bridge connecting the daughter cells in 2-cell embryos (Fig. 7F'). In the activated eggs by the sperm from another species males, only female pronucleus undergoes genome replication and completes the first cleavage (Fig. 7A''–F''), which is typical gynogenesis, whereas no any sperm nucleus replication signal is visualized during the whole first mitosis (Fig. 7A''–F''). Significantly, the BrdU incorporated female pronuclei in later two kinds of fertilized eggs (Fig. 7A',7A'') are bigger and displays more intensive fluorescence than that in the former zygote development eggs (Fig. 7A), and there exists the first mitosis metaphase delay (Fig. 7C',7C'') compared to the zygote development eggs (Fig. 7C).

## Discussion

**Figure 8:**
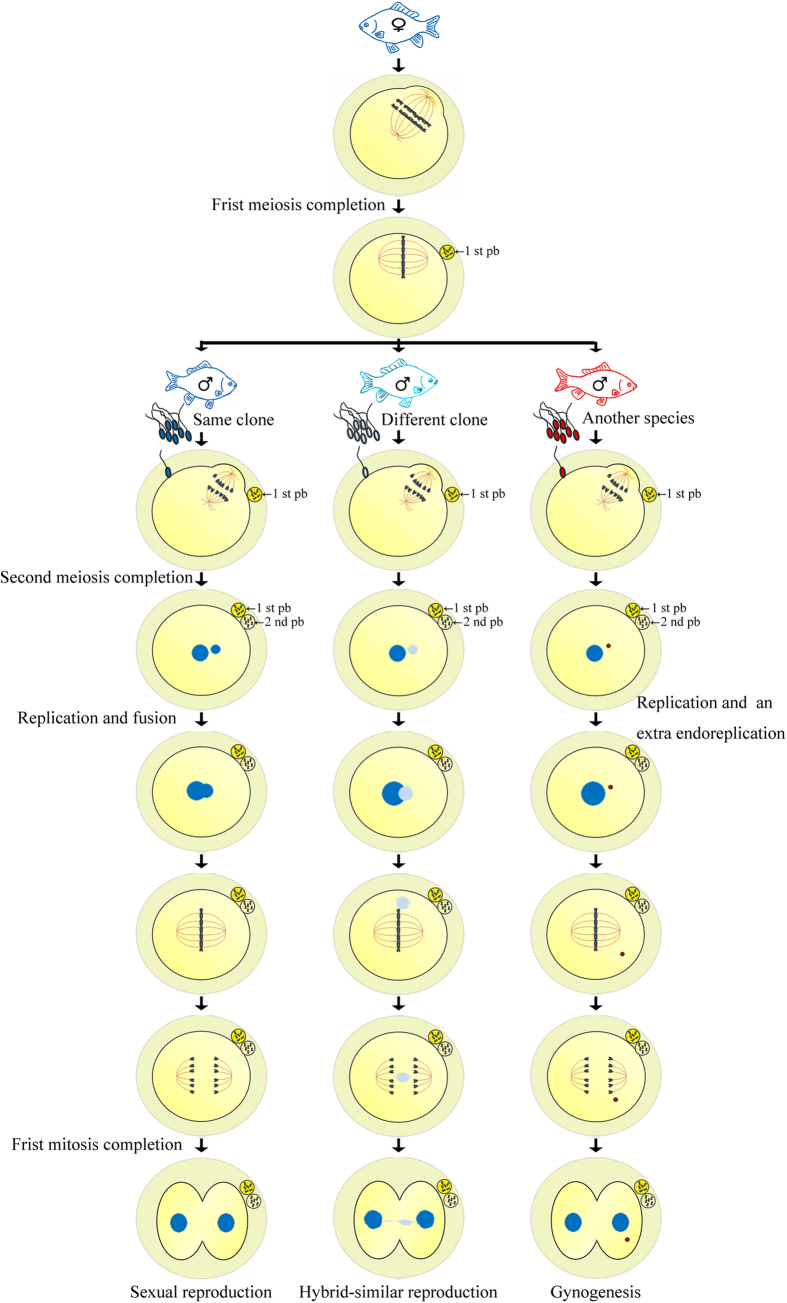
Schematic diagram of normal meiosis completion and various sperm response reproduction modes. (Left) Sexual reproduction mode in response to the sperm from the same clone male. (Middle) Hybrid-similar reproduction mode in response to the sperm from different clone male. (Right) Unisexual reproduction mode of gynogenesis in response to the sperm from another species male. Different sperm nucleus and the corresponding male pronucleus are shown by the corresponding colors. 1st pb and 2nd pb indicate the first polar-body and the second polar-body. The main key steps during meiosis and the first mitosis, including first meiosis completion, second meiosis completion, genome replication and fusion, genome replication, and a hypothesized extra endoreplication, are indicated in the corresponding positions. All the images of fish were drawn by Jun Zhang and scanned into digital pictures.

The above data indicate that clone D completes normal meiosis because the meiotic pachytene bivalents are just about half of somatic chromosomes (Fig. 3), and extrudes the first polar body (Fig. 2 and Fig. 4) and second polar body (Fig. 5) along with the oocyte maturation and egg fertilization. And, three types of various nuclear events and development behaviors have been observed in the fertilized eggs by three kinds of different sperms (Fig. 6), and confirmed by BrdU incorporation-marked immunofluorescence detection (Fig. 7). Therefore, these findings suggest that clone D should undergo normal meiosis completion, including two successive meiotic divisions and two polar body extrusions, during oocyte maturation and egg fertilization, and the mature egg should possess three various sperm responses for the unisexual and sexual reproduction modes. Figure 8 shows the schematic diagram. The mature oocyte completes the first meiosis and extrudes the first polar-body during the oocyte maturation. Along with sperm enters, the mature egg further completes the second meiosis, extrudes the second polar-body, and starts various response reproduction modes. In response to the sperm from the same clone male, it is a typical sexual reproduction mode in which the male pronucleus and female pronucleus fuse to form the zygote and then to undergo the first mitosis. In response to the sperm from another species male, it is a typical unisexual reproduction mode of gynogenesis, in which only female nucleus develops to enter the first cleavage^6^,^8^. In response to the sperm from different clone male, the currently observed unusual chromosome behaviors including the divorced paternal chromatin (Fig. 7D') and the resulted chromatin bridge (Fig. 7F') are very similar to that reported previously in interspecific hybrid^50^. So, we call it as a hybrid-similar development mode. Based on their nuclear behavior differences and the first cleavage completion delay, we hypothesize that an extra pre-cleavage endoreplication might occur in the gynogenetic response mode and in the hybrid-similar response. Significantly, these current findings highlight several interesting evolutionary issues on reproduction success in polyploid vertebrates, such as the evolutionary significance of meiosis completion and the evolutionary consequences of reproduction mode diversity.

### Evolutionary significance of meiosis completion in polyploid vertebrates

As meiosis ensures genome stability and creates genetic diversity in all sexual diploid organisms^51^, polyploid species have been proposed to be very difficult to complete normal meiosis and meiotic recombination owing to the polyploidy with more than 2 sets of genomes. In unisexual polyploid vertebrates, several meiosis deviations, including oogonial fusion^14^, premeiotic endoreplication^14^,^37^,^38^,^39^,^40^, apomixes or ameiosis^41^, have been observed to generate the unreduced eggs, and gynogenesis and parthenogenesis have been demonstrated to reproduce viable offspring that carry all chromosomes of the mother genomes and therefore are clones of the mother^8^,^14^. Interestingly, our current studies have not only observed normal meiosis completion, and but also found various sperm response mechanisms for the unisexual and sexual reproduction modes in the hexaploid clone D. Perhaps, it is the meiosis completion and various sperm responses that lead to the multiple modes of unisexual gynogenesis and sexual reproduction.

Actually, normal meiosis and sexual reproduction were also observed in green toads (*Bufoviridis* subgroup)^52^ and water frogs (*Pelophylax esculentus*)^53^ with various genome ploidy levels. And, simultaneous Mendelian and clonal genome transmission was further revealed in sexual reproduction all-triploid toads^54^. In addition, two symmetric allotetraploid populations were reported to resume normal meiosis after undergoing intermediate processes of non-sexual reproduction and thereby to lead to a new sexually reproducing polyploid in a small cyprinid fish *Squalius alburnoides* complex that are composed of diploid hybrid, triploid and tetraploid forms^55^. In *Misgurnus* loach complex with diploids, triploids and tetraploids, some clonal diploid lineages were also found to reproduce by gynogenesis, and such clonal diploid loach hybrids were able to generate the unreduced diploid eggs through a premeiotic endoreplication mechanism^56^. Similarly to the currently analyzed *Carassius gibelio*, all of the above polyploid lineages are allopolyploids, which imply that a similar evolutionary way might have occurred in these polyploid animals. However, in contrast with the above other polyploid lineages, the reduced eggs generated from normal meiosis in the clone D are able to perform both of sexual reproduction and unisexual gynogenesis, and the various development initiation only depends the entered sperm. As reported previously by us^8^,^23^,^30^, the hexaploid *Carassius gibelio* might originate from the already diploidized tetraploid, and diploidization should have continually proceeded. As an ongoing or nearly diploidized polyploid, clone D has evolved into normal meiosis. It is the meiosis completion that further develops and maintains the multiple reproduction modes, including sexual reproduction and unisexual reproduction. A recent finding from genome duplicated plant (*Arabidopsis arenosa*) has revealed that adapting meiosis is a necessary step on the way to becoming a successful polyploid^57^. Therefore, the meiosis completion has significant implications for the reproduction success and evolutionary potentials of polyploid vertebrates.

### Evolutionary consequences of reproduction mode diversity in polyploid vertebrates

Another intriguing issue in this study is about the finding that there are various sperm responses to the deduced eggs, and it is the various sperm responses that lead to the multiple modes of unisexual gynogenesis and sexual reproduction. In fact, a recent genetic investigation on several wild populations of gibel carp has confirmed the ecological roles and evolutionary consequences, because the positive relationship between a specific mtDNA haplotype proportion in the populations and its abundance in the males has been revealed, which implies that sexual reproduction might play critical roles in population amplification, and might also exert significant function in creating genetic diversity^30^. Theoretically, sexual reproduction can lead to genetic diversity and generate fit genotypes, whereas unisexual gynogenesis can quickly amplify them. Therefore, the various sperm responses and the caused multiple modes of unisexual gynogenesis and sexual reproduction might create diverse clones with significant genetic difference^58^ and allow them to rapidly adapt and colonize various habitats. Actually, a large number of various clones with different genetic background have been discriminated from polyploid *Carassius gibelio*, and all of the studied clones possess the ability of unisexual gynogenesis^8^. Whether these various clones also complete normal meiosis and possess multiple reproduction modes or some of them only possess unisexual reproduction mode will be worthy of further and deep investigation.

Almost in all polyploid vertebrates, unisexual forms generally coexist with the sexual species. And, in these sexual-unisexual complexes, only one unisexual reproduction mode, such as gynogenesis, parthenogenesis, or hybridogenesis, is adopted by the unisexual forms^59^. As suggested previously by us, the coexisting multiple modes of unisexual gynogenesis and sexual reproduction in polyploid *Carassius gibelio* should an indicative case of reproduction mode diversity evolution from unisexual reproduction towards sexual reproduction^34^. Therefore, the coexisting multiple modes and the underlying control mechanism will provide us a special case to explore sexual origin and sex evolution in vertebrates.

## Conclusion

Unisexual vertebrates are known to reproduce by gynogenesis, parthenogenesis, or hybridogenesis, but their cytological and developmental mechanisms have remained unclear. This study provides direct evidence for explaining the reasons why unisexual and sexual reproductions are able to coexist in clone D of polyploid *Carassius gibelio*, because its mature eggs have completed normal meiosis, and have three various development modes, such as sexual reproduction in response to the same clone sperm, unisexual gynogenesis to another species sperm, and hybrid-similar development mode to another different clone sperm.

## Materials and methods

### Collection of oocytes and embryos

Clone D and clone F in polyploid *Carassius gibeli* as well as red common carp (*Cyprinus carpio*) used in this study were maintained and obtained from Guanqiao Experimental Station, Institute of Hydrobiology, Chinese Academy of Sciences. During the reproduction season, the selected brood fish of clone D were artificially induced into spawning by intraperitoneal injections with a mixture of acetone-dried carp pituitary, HCG, and LRH-A. Different stage oocytes were collected by a self-made egg-taker every 2 h from the injection until the moment of mature spawning. The ovulated eggs were inseminated by three kinds of different sperms from the same clone (clone D) males or different clone (clone F) males or from another species red common carp as described previously^49^. The fertilized eggs were incubated at about 23 °C for cytological observations. All experiments in this research were performed according to the permit guidelines established by the Institute of Hydrobiology, Chinese Academy of Sciences, and the experimental protocols were approved by the animal care and use committee of Institute of Hydrobiology, Chinese Academy of Sciences.

### Immunofluorescence localization

Oocytes or mature eggs were collected and freshly fixed for 6–8 h by MSB (microtubule-stabilizing buffer, 80 mM K-PIPES, 5 mM EGTA, 1 mM MgCl2, 3.7% PFA, 0.25% glutaraldehyde, pH 7.4) at room temperature. After the oocytes or eggs were sectioned at 10 μm in thickness with frozen microtomy (Leica), they were incubated with primary antibody (anti-β-tubulin antibody) and secondary antibody (Rhodamine-conjugated goat anti-mouse antibody). Finally, propidium iodide (PI) or DAPI were employed for nuclei staining. The slides were imaged on Laser scanning confocal microscope (Zeiss LSM710) or Leica fluorescence microscope. All details were described previously^45^,^49^.

### Meiotic pachytene chromosome preparation in oocytes

The collected oocytes from mature females were placed in PBS, and the germinal vesicles (GVs) were isolated using fine forceps according to a previous report in lizards^60^. Then, GVs were transferred with a pipette to glass-bottom dishes containing MSB (pH 7.4) and allowed to spread and fix for 1 h at room temperature. Moreover, the prepared meiotic pachytene spreads were stained by DAPI for 30 min and visualized under two photon of Laser scanning confocal microscope (Zeiss LSM710).

### BrdU incorporation and immunofluorescence detection

BrdU (5-bromo-2-deoxy-uridine) incorporation assay was performed as described previously^49^. In brief, eggs were microinjected with BrdU within 10 min after fertilization. Then BrdU incorporation embryos were fixed and permeabilized. After treating with 2N HCl, the embryos were subjected to α-BrdU antibody staining. Immunofluoresecence imaging was performed by Laser scanning confocal microscope (Zeiss LSM710).

## Additional Information

**How to cite this article**: Zhang, J. *et al*. Meiosis completion and various sperm responses lead to unisexual and sexual reproduction modes in one clone of polyploid *Carassius gibelio*. *Sci. Rep*. **5**, 10898; doi: 10.1038/srep10898 (2015).
